# Odontological Society of Pennsylvania

**Published:** 1890-07

**Authors:** 


### ODONTOLOGICAL SOCIETY OF PENNSYLVANIA.

The regular meeting of the Odontological Society of Pennsylvania was held Saturday evening February 1, 1890, at the hall, Thirteenth and Arch Streets. President Truman in the chair. An address on the "Degeneracy of Tooth-Structure" was delivered by Harrison Allen, M.D., who spoke as follows:

I desire, in the first place, to speak of the manner in which the word degeneration will be used in connection with tooth-form. I will attempt to show that degeneration is a form of specialization. Degeneration, therefore, can never be seen in a tooth which is not highly specialized. A degenerated tooth is more apt to appear towards the end of the series to which it belongs than at the beginning. Conceding that the molars of the upper jaw form a true series, degenerations are more frequently seen in the second and third than in the first tooth. The same remark can be applied to all organic structures which are arranged in series. Professor Alpheus Hyatt (On the Genesis of the Arietidæ) has called attention to the degenerations which occur in the last-formed chambers of Ammonites; these are not different in kind from those in the molar series. An apparent explanation for degenerative changes lies in the fact that the structures which are formed last are at the same time part of a natural succession, and, having a common origin, tend to weaken as they appear at the end.

A degenerated tooth is distinguished from others by the thickening of the lateral contour-lines. If a tooth (like the bicuspid) is composed of two cusps arranged from the buccal to the palatal surface, the lateral contour-lines are those which connect the anterior and posterior borders of the cusps with each other. A tooth in advanced degeneration may exhibit the lateral contour-lines in such proportions as to equal in height the true cusps, and the tooth may thus appear to be composed of a single cusp which is excavated and truncated. Such a form of tooth may receive the name of crater-like tooth. It is often met with in the human third molar.

A general form of degeneration is seen in the molar teeth, especially in those which vary by the reduction of the normal number of cusps. It is a mistake to assume that when cusps are lost (as, for example, a "four" cuspidate form becoming a "three" cuspidate) the tooth is always an example of the principle of reversion. The cusps may be lost by degenerative process, and no reversion be suggested.

It is evident that, in order to have a reversion, one must demonstrate the form of which the reversion occurs. If I were to take as examples the human teeth and name the variations in their forms on the principle of reversion, I would suggest a motley group of structures,—strange company, indeed, for man and his kinship to keep! For instance, I would bring together ape-like structures, as those seen in Cebus, Alouatta, Lemur, and Cercopithecus; hedge-hog-like structures, as in Erinaceus and Centetes; bat-like and mole-like, as in Atalapha and Talpa; to say nothing of forms suggestive of those of sirenians and rodents, as well as those of ancestry as remote as those of Phenacodus, Peritychus, and Coryphodon, which Professor Cope has told us so much about. Such an insistence is absurd. No; many of the variations in the human jaws are not reversions but degenerations; that is, they are failures on the part of a majority of instances of a given type-form to keep up to the standard. It may be asked, "Are not your degenerations mere monstrosities?" I reply that they are not, since they are consistent within themselves,—are confined to lines within those suggested and maintained elsewhere in the dental series,—they are never eccentric, and always suggest the normal relation of parts as these are seen in the lower animals.

There is a degenerate form of human tooth-structure which is exhibited in the first and second of the series of the upper jaw, which I have ventured to call the *ellipsoid form*, and describe it as follows:

The ellipsoid tooth is a conversion of the quadrate to the ellipsoid shape of crown. It is an instance worth mentioning that all the ellipsoidal teeth which I have had extracted from the jaw were found, on examination, to be coalescent at the roots. Out of fifteen examples which I have recently examined, six were complete in coalescence; in a word, all three roots were merged into one. In seven, the two buccal or ectal cusps had coalesced; in the remaining two, while all three were distinct, the ectal or palatal cusps were closely proximate in their entire length instead of being divergent as is the rule. It is evident that in this form of tooth less resistance is afforded for the tooth to retain its position in the jaw than would be the case if the roots were widely divergent.

In the living subject I have frequently observed the ellipsoid form of molar tooth, and I have never in a single instance found it in a jaw in which the teeth were of the best type. Very commonly it is the second molar (although it may be the first) which exhibits this peculiarity, and the third molar will show a markedly degenerated simple form of tooth. The ellipsoid tooth is a witness to the weakness in the original germ for the molar series.

Professor Cope replied to Dr. Allen's remarks as follows:

I agree in part and in part disagree with Dr. Allen. I was glad to hear him draw the distinction between pathological and biological degeneration. With reference to the tri-tubercular superior molar of man, I still adhere to my opinion that the type of the superior molar is a reversion to the molar of the primitive mammalia; and since the Lemuridæ are the latest type which displays this tooth in the phylogeny of man, I would call it the lemurine reversion, and the lemurine molar. I think this position is self-evidently correct, and that the molar in question is not a monstrosity. The obtuse form of the cusps is not monstrous but degenerate, and is identical with the modification which takes place in the true molars and the sectorials of the carnivora, where they are similarly tri-tubercular.

Such modification is to be seen in the genus Paradoxurus in the bivenidæ; the sea otters and badgers in Mustelidæ; and the bears in Canidæ.

Professor Cope exhibited the cranium, with molars preserved, of a Tahitian Islander, in which the third superior molar on one side was tri-tubercular, and on the other side monstrous. He also exhibited crania of two species of Paradoxurus, in which the superior molars greatly resembled the human tri-tubercular type. He agreed with Professor Allen that a great many monstrous molars are met with in the dentition of civilized races of man.

